# Deciphering the Photocatalysis Mechanism of Semimetallic Bismuth Nanoparticles

**DOI:** 10.1021/acs.jpcc.4c06136

**Published:** 2024-11-16

**Authors:** Lauren M. Hoffman, Delaney J. Hennes, Pin Lyu

**Affiliations:** Department of Chemistry and Biochemistry, University of North Carolina Asheville, 1 University Heights, Asheville, North Carolina 28804, United States

## Abstract

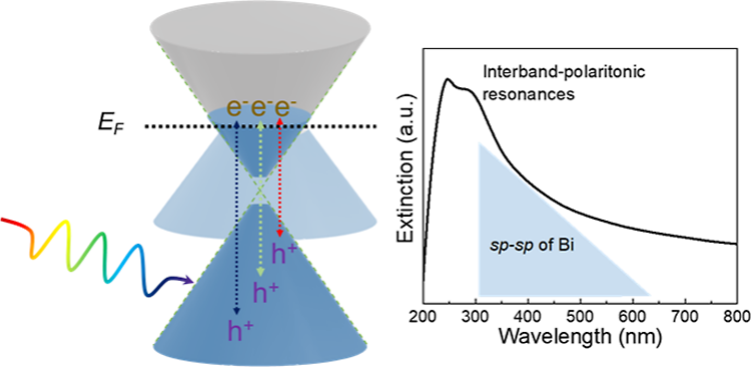

Metallic nanoparticle photocatalysts have been developed in various catalytic systems over the past few decades, including diverse noble and non-noble metals with plasmonic properties. The hot-carrier-induced mechanism is one of the most appealing pathways as it can provide energetic electrons or holes for driving thermodynamically unfavorable reactions or increasing the reaction rate. In this work, we evaluate the photocatalytic performance of semimetallic bismuth nanoparticles and offer detailed mechanistic interpretations in terms of hot carriers and interband transitions. The photocatalyzed nitrophenol reduction with sodium borohydride serves as a model reaction, and a wavelength-dependent study reveals the contribution of hot carriers. It is demonstrated that light irradiation under shorter wavelengths could produce deeper hot holes in bismuth nanoparticles, which can be quenched more effectively by hole scavengers, thus facilitating the electron-transfer process and resulting in larger apparent reaction rate constants. The observed photocatalysis enhancement accounts for the unique band structure with an extremely small band gap and exclusive interband absorption in the visible region. This proof-of-concept work offers a different perspective on the photocatalysis mechanism of bismuth nanoparticles and could help us better understand the role of hot carriers involved in photocatalysis, especially with interband transitions.

## Introduction

Metallic nanoparticle photocatalysts have emerged with profound interest in fundamental research and practical applications for the past decades.^1−14^ The unique and tunable optical properties, various light-assisted reaction pathways, and robust nature of coupling with other photoactive materials are among the most significant potentials. While precious metals such as Au and Ag have been extensively studied as representatives of plasmonic nanoparticle photocatalysts and have demonstrated promising reactivities toward various reactions, high material cost still hinders their large-scale industrial operations.^15,16^ Thus, exploring non-noble-affordable alternatives for metallic nanoparticle photocatalysts and deciphering their photoexcitation mechanisms is of great necessity.

Several non-noble metals have been identified as promising candidates for photocatalysis, including transition metals (copper,^17−20^ nickel,^21−23^ and cobalt^24,25^), post-transition metals (aluminum^26−30^ and bismuth^31−33^), and others.^15,16^ One example is that Linic and co-workers reported visible-light-enhanced propylene epoxidation selectivity mediated by localized surface plasmon resonance of copper nanoparticles and by photoinduced switching of Cu oxidation states during reactions.^17^ Wang, Zeng, and co-workers discussed recent advances and future perspectives of Cu-based plasmonic nanoparticles in this review.^19^ Another example is that Carter, Nordlander, Halas, and co-workers demonstrated the hot-electron-induced hydrogen dissociation by LSPR and direct interband transitions of Al nanocrystals under ambient conditions.^34^ Halas and co-workers highlighted the recent progress of Al nanocrystals in this account.^28^ Meanwhile, less research has been emphasized on Bi nanoparticles for photocatalysis,^31−33^ especially in the colloidal free-standing form, which is more convenient and ideal for mechanistic interpretation. Considering the small carrier effective mass, long carrier mean free path, and high carrier mobility in the bulk version,^35−37^ Bi nanoparticles could potentially leverage these advantages to be a better photocatalyst.

Regarding the photoexcitation and catalysis mechanisms of metallic nanoparticle photocatalysts, multiple pathways could be involved, such as hot-carrier generation and transfer,^38^ resonance energy transfer,^39^ near-field enhancement,^40^ photocharging,^41^ and photothermal effect.^42^ Concerning the photocatalyzed model reaction of 4-nitrophenol reduction employed in this work,^43^ the first mechanism is our focus, and the energetics and dynamics of these hot electrons/holes highly depend on the electronic structure of different nanoparticles.^44^ Two predominant sources of these hot carriers are intraband (from LSPR) and interband (from LSPR-decay or direct) transitions, detailed in our recent featured article.^14^ The rationale for utilizing interband transitions to shift the nanoparticle photocatalysts from noble to non-noble metals is their ubiquitousness, less influenced by geometric factors like size and shape.^14^ More importantly, the deep holes with high oxidation potential generated from interband transitions provide an unprecedented opportunity for mechanism exploration, which has been proven beneficial for many photocatalytic reactions recently.^45−50^

**Scheme 1:**
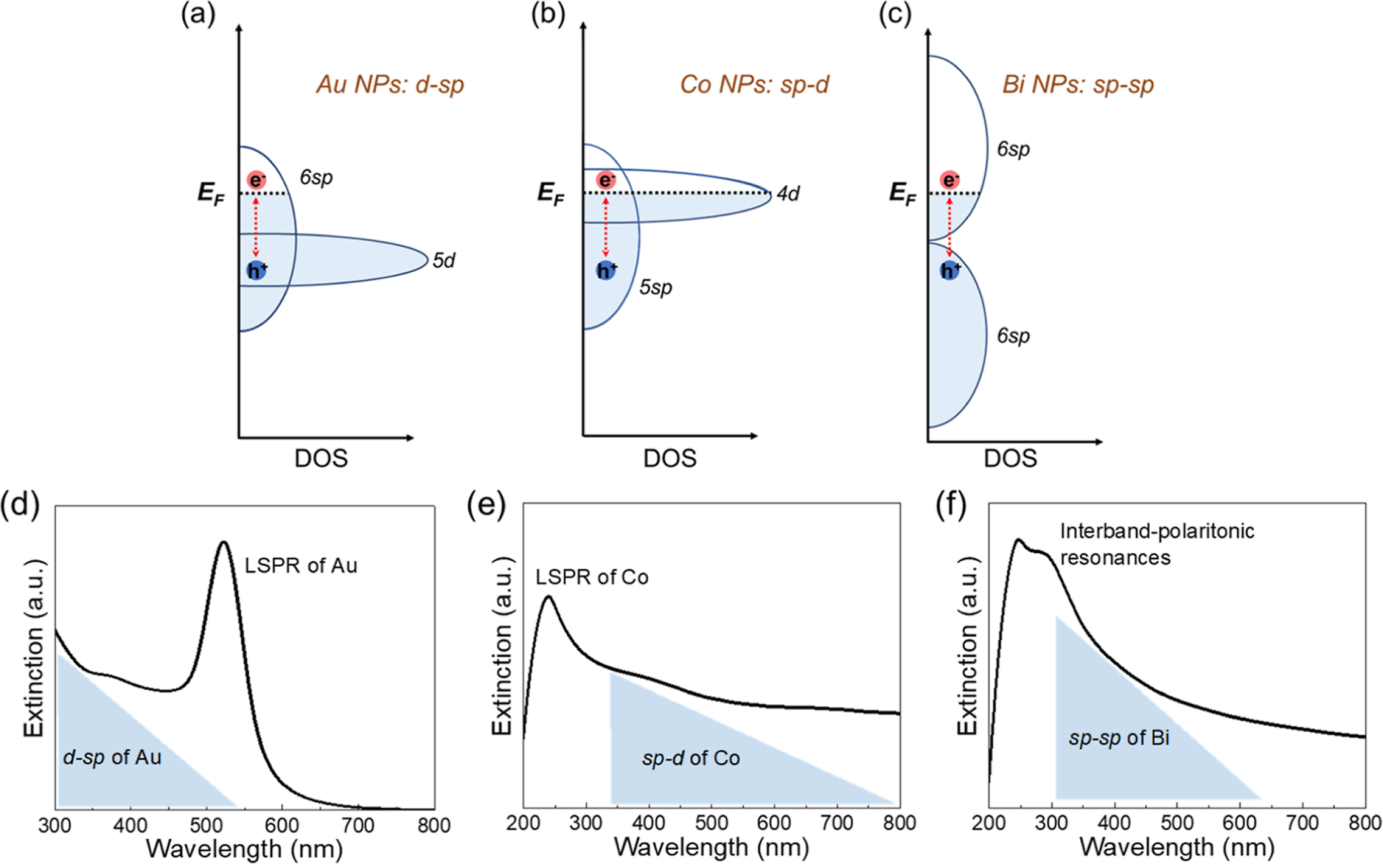
Interband Transitions and Typical Extinction Spectra of Different Metallic Nanoparticles: (a,d) Au Nanoparticles, 5d-6sp Electronic Transitions with a Cutoff Threshold of 520 nm, LSPR of 40 nm Spherical Nanoparticles around 522 nm; (b,e) Co Nanoparticles, 5sp-4d of 345–4100 nm, LSPR of 70 nm Co Nanoparticles around 240 nm; and (c,f) Bi Nanoparticles, 6sp-6sp of 310–620 nm, Interband-Induced Localized Surface Plasmon-like Resonances around 280 nm *E*_F_ represents the Fermi level, and to cross-compare, all *E*_F_ levels are fixed at the same level in the scheme. LSPR for localized surface plasmon resonance.

In this work, we explore the photocatalytic performance of colloidal Bi nanoparticles and correlate the photoexcitation mechanisms and hot-carrier behavior based on their unique semimetallic band structures. As shown in Scheme 1, moving from noble Au to non-noble Co nanoparticles, the less-filling of d electrons makes the Fermi level cross over the d-band and less oscillation of free electrons, thus shifting the plasmon resonance to a shorter wavelength (mainly in the deep-UV region).^51,52^ At the same time, interband transitions still spread across the visible spectrum but vary from d-sp transitions of Au to sp-d transitions of Co.^53,54^ The systematic comparison of photocatalysis mechanisms between these two metals was just reported in our recent publication.^25^ Furthermore, bismuth, as a semimetal with a highly anisotropic Fermi surface (three *L*-point electron pockets and one *T*-point hole pocket), has an exceptionally small direct band gap of 36 meV.^55,56^ While the free-carrier-oscillation-induced plasmonic response of Bi is expected in far-IR to terahertz (THz), the strong interband transitions in the UV–visible region will induce resonance in Bi nanostructures, defined as “interband plasmonic resonance”.^37,57−59^ At least three interband transitions have been identified to be contributing to the strong optical absorption in this region both theoretically and experimentally, including Γ_6_^+^–Γ_6_^–^ and Γ_45_^+^–Γ_6_^–^ near Γ point, and *T*_6_^–^–*T*_45_^–^ near *T* point.^37,60,61^ The photogenerated hot carriers (electron–hole pairs) resulting from these strong interband absorptions could be extracted and transferred to the metal–adsorbate hybridized states, thus enhancing the catalytic activity of the Bi nanoparticles.

As follows, our experimental approach to validate the interband transitions as the photocatalysis mechanism of Bi nanoparticles is by wavelength-dependent studies in the surveyed absorption region and by comparing the initial reaction rate constants from chemical kinetics. Considering the short lifetime of hot holes (about tens of femtoseconds)^62^ and the relatively long time interval between two absorbed photons (about tens of nanoseconds),^45^ our strategy is to exploit hole scavengers to quench the hot holes for better electron extraction or for charging the nanoparticles with accumulated electrons for catalysis. The photocatalyzed 4-nitrophenol reduction reaction with sodium borohydride serves as the model reaction to provide kinetics analysis. Eventually, the photocatalysis mechanism of interband transitions in Bi nanoparticles is discussed and aligns well with the trend of better photocatalyzed reaction rate enhancement at shorter wavelengths. These findings shed light on the mechanistic insight into the semimetallic bismuth nanoparticle photocatalysts and are expected to intrigue some new strategies toward designing more affordable photocatalysts.

## Experimental Methods

### Chemicals and Characterizations

All chemicals and reagents were used without any purification. Reaction kinetics were monitored by using an UV–vis spectrometer (Ocean Optics HR2). The morphology of Bi nanoparticles was examined by transmission electron microscopy (TEM, Talos F200C G2, 200 kV, Thermo Fisher Scientific) and scanning electron microscopy (SEM, JSM-IT700HR, 15 kV, JEOL). The size distribution and zeta potential in reaction solutions were measured by dynamic light scattering (DLS, Zetasizer Pro, Malvern Panalytical). The local crystalline structure of Bi nanoparticles was determined by powder X-ray diffraction pattern (PXRD, Bruker D2 PHASER Benchtop XRD, Cu tubes, 30 kV, 10 mA) and was processed with DIFFRAC.EVA data analysis software with the Crystallography Open Database (rev. 278581). The surface oxidation state of Bi nanoparticles used before and after catalyzed reactions was determined by X-ray photoelectron spectroscopy (XPS, Nexsa, Al Kα X-ray source, Thermo Fisher Scientific), in which the contamination carbon (C–C) around 284.9 eV was set as the reference to calibrate the binding energy of other elements. The samples were prepared by drop-casting and drying the Bi nanoparticle solution on a silicon wafer and sent for XPS immediately.

### Synthesis of Bi Nanoparticles

The synthesis procedure was modified from the water-based chemical reduction method developed by Zhao and co-workers.^63^ Briefly, 2 mmol bismuth(III) chloride (BiCl_3_), 31.5 mg of sodium oleate (NaOl, weight ratio to BiCl_3_ is about 1:20, serving as the stabilizing capping ligands for nanoparticles), and 20 mL of hydrazine hydrate (N_2_H_4_, 55% in water) were mixed under rapid stirring for 30 min. The mixture was heated to 80 °C in a water bath for 60 min and ripened for 10 min before washing with a 0.5 mM sodium oleate solution three times. The washing procedure was followed by centrifugation at 7000 rpm for 10 min, discarding the supernatant solution, and resuspension in 0.5 mM NaOl solutions. The final as-synthesized Bi nanoparticles were stored in 0.5 mM sodium oleate solution for further characterization and catalysis reactions.

### 4-Nitrophenol Reduction with Bi Nanoparticles

In a typical reaction condition, 750 μL of 4-nitrophenol (0.2 mM in H_2_O), 750 μL of freshly made sodium borohydride (NaBH_4_, 60 mM in H_2_O), and 800 μL of H_2_O were mixed in a quartz cuvette (1 × 1 cm, R-3010-T, Spectrocell) under constant stirring. Then, 200 μL of Bi nanoparticle stock solution (93.8 mM in 0.5 mM NaOl solution) was added into the cuvette. The time-dependent UV–vis profiles were recorded every 2.5 min for the first 15 min. The absorbance at 400 nm was assigned to 4-nitrophenolate (the deprotonated form of the reactant from mixing 4-nitrophenol with NaBH_4_) and calibrated by subtracting the background absorbance of Bi nanoparticles. The reduction product peak at 268 nm was assigned to 4-aminophenol (4-AP). The isosbestic point at 312 nm was observed to confirm the successful reduction reaction. The corresponding concentration of nitrophenolate (*C*_*t*_) based on Beer’s law was fitted to the linear plot of ln (*C*_*t*_/*C*_0_) vs time to extract the apparent reaction rate constant *k*_app_.

As for the photocatalyzed reactions, 20 μL of isopropanol (IPA) as the hole scavenger was added into the cuvette, and different light-emitting diodes (LEDs, with center wavelengths at 405, 415, 455, and 530 nm, Thorlabs) were used to photoexcite the Bi nanoparticles. A cooling fan was used to keep the reaction at room temperature, and the rest of the conditions were the same as the nonirradiation reactions above unless specified. The incident power was measured by a USB power meter (PM16-121 with a Standard Photodiode Sensor, Thorlabs). Considering the spectral overlap between the 4-nitrophenolate and the LED sources, control experiments without Bi nanoparticles under different wavelengths and power were conducted to exclude the contribution of photodegradation from the photocatalysis of Bi nanoparticles.

### Control Experiments with Bi_2_O_3_ Nanoparticles for 4-Nitrophenol Reduction

The bismuth(III) oxide nanopowder was purchased from Thermo Fisher Scientific Inc. with an average size of 80–200 nm. The stock solution of Bi_2_O_3_ nanoparticles was prepared in 0.5 mM NaOl solution with a molar concentration of about 2.58 mM to maintain a similar absorbance to the Bi nanoparticles. 200 μL of Bi_2_O_3_ nanoparticles stock solution was added into the reaction solutions with all other conditions remaining the same as the reaction with Bi nanoparticles above.

## Results and Discussion

**Figure 1:**
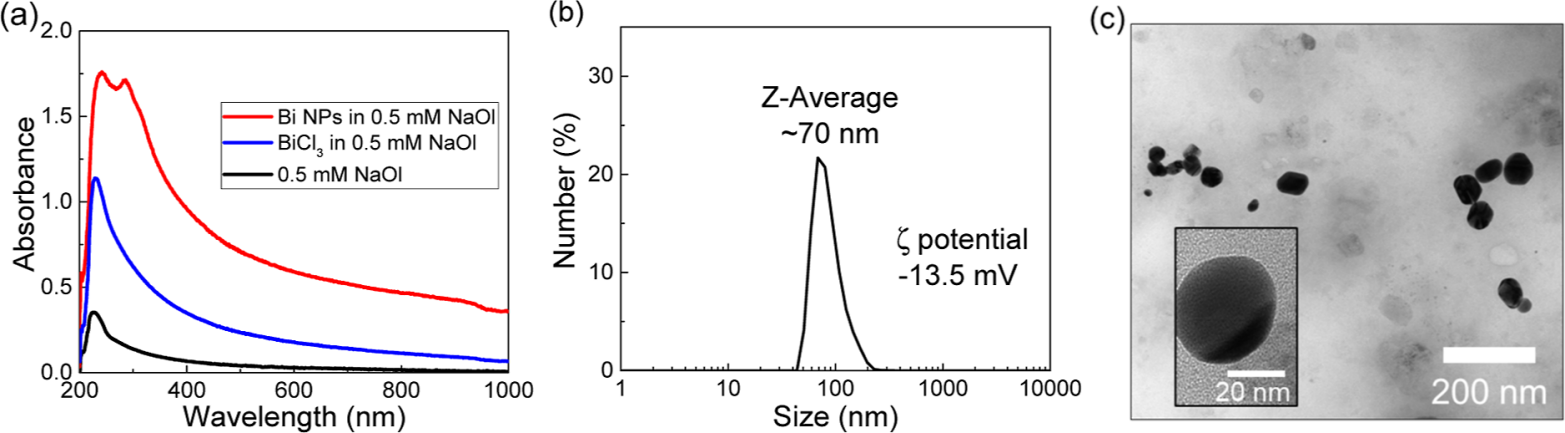
Optical properties and size distribution analysis of Bi nanoparticles. (a) UV–vis spectra of Bi NPs and BiCl_3_ in 0.5 mM NaOl (sodium oleate) solution. (b) Hydrodynamic diameter and zeta potential of Bi NPs in 0.5 mM NaOl in number percentage measured by DLS. (c) TEM image of Bi NPs with a scale bar of 200 nm and one representative Bi nanoparticle in the inserted high-resolution image with a scale bar of 20 nm.

The Bi nanoparticles were synthesized by a water-based chemical reduction method with BiCl_3_ as the precursor, NaOl as the capping ligand, and N_2_H_4_ as the reducing agent.^63^ The nanoparticles were reported to be stable without obvious oxidation for at least six months, and we dispersed them in 0.5 mM NaOl solution for further characterization and catalysis. The colloidal form of the Bi nanoparticles is confirmed in Figure S1 and regularly checked before using them as photocatalysts. The UV–vis absorbance in Figure 1a demonstrated that the extra strong absorption peak at around 280 nm belongs to the interband-induced plasmonic resonance, as discussed in the Introduction section. The intrinsic direct interband transitions range from 310–620 nm^15,59^ as their optical response decreases with lower photon energy. The Bi nanoparticle size distribution was analyzed by DLS with an average of 70 nm in hydrodynamic diameter in NaOl solutions in Figure 1b and by TEM with a range of 43 ± 7 nm in the dried powder form in Figures 1c and S2. The as-synthesized nanoparticles are not uniformly spherical; however, as mentioned above, the interband transitions as intrinsic electronic transitions mainly depend on the band structures of the metal and are less influenced by geometric factors. As long as the nanoparticles are well-dispersed colloidally in the reaction solutions (observed under all of our reaction conditions), the reaction rate constants discussed below are still the most reliable indicators for comparing different photocatalyzed reactions, especially across different wavelengths.

**Figure 2:**
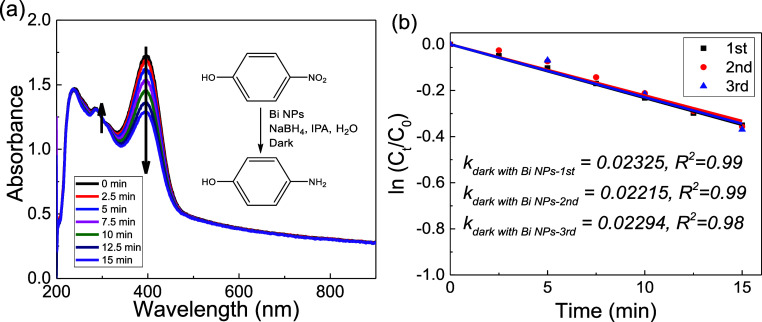
4-Nitrophenol reduction with Bi NPs and NaBH_4_ as a model reaction. (a) Representative UV–vis time-dependent profile and (b) corresponding kinetics analysis of three replicated reactions under nonirradiation conditions for initial apparent rate constants extraction. The linear fits follow a pseudo-first-order process for 4-nitrophenol.

The reduction of nitroaromatic compounds in aqueous solutions is often chosen as a model reaction to test the catalytic performance of metallic nanoparticles,^43^ as well as in photocatalyzed reactions.^64,65^ The 4-nitrophenol reduction with sodium borohydride to 4-AP was employed for the Bi nanoparticles under nonirradiation conditions, as shown in Figure 2a. The time-dependent UV–vis profile was recorded by UV–vis spectroscopy, and the concentration of reactants was calculated by the difference of absorbance at 400 nm (in the form of 4-nitrophenolate ions) and the background absorbance of the Bi nanoparticles. The reduction product peak at 268 nm was assigned to 4-AP, and the isosbestic point at 312 nm was observed to confirm the successful reduction reaction (Figure 2a). The 4-nitrophenol reduction does not proceed without Bi nanoparticles, without NaBH_4_, or without both in Figure S3, confirming the catalytic activity from the Bi nanoparticles and excluding other possible reaction pathways.

The commonly accepted 4-nitrophenol reduction reaction mechanism is described by the Langmuir–Hinshelwood model with both nitrophenol and surface-hydrogen species (from borohydride ions) adsorbed on the nanoparticle surface, and the reduction of intermediate 4-hydroxylaminophenol to final product 4-AP is considered as the rate-determining step.^66,67^ Considering the excess amount of NaBH_4_ used in our reaction condition (molar ratio of 300 times to 4-nitrophenol), a pseudo-first-order analysis on reactant 4-nitrophenol (4-nitrophenolate by absorbance) is applied to extract the apparent reaction rate constant (*k*_app_), as shown in Figure 2b. It is noteworthy that we did not observe the induction time of the 4-nitrophenol reduction for Bi nanoparticles, whereas much slower kinetics were detected right at the beginning of the reactions, as is common in other oxidizable or nonoxidizable metallic nanoparticles.^68,69^ We speculate that relatively high borohydride concentration in the solution and relatively low oxygen concentration in the sealed cuvette could result in rapid reconstruction of the surface or removal of the surface oxide layer of Bi nanoparticles.^68,70^

**Figure 3:**
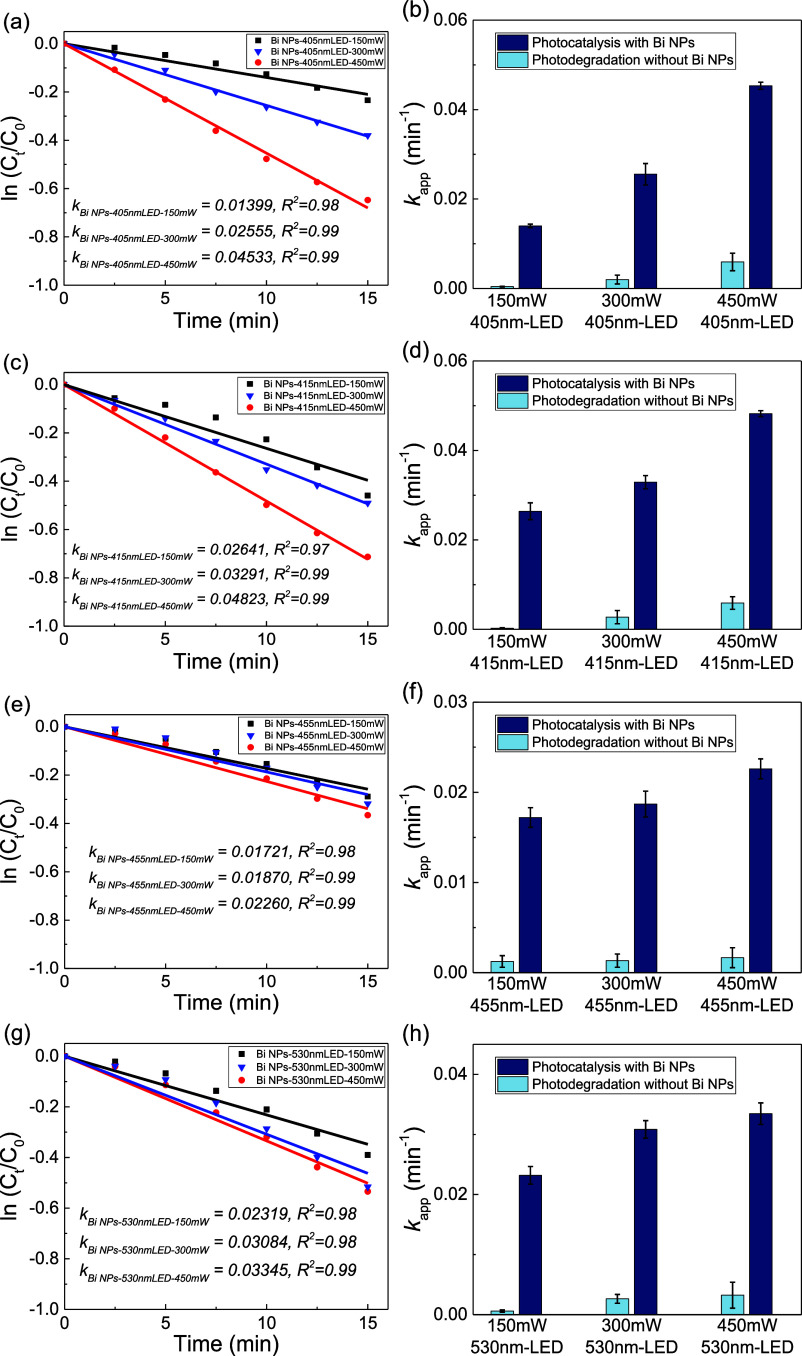
Photocatalytic performance of Bi NPs under different center wavelengths and power intensities of LED irradiations. (a,b) 405 nm LED, (c,d) 415 nm LED, (e,f) 455 nm LED, and (g,h) 530 nm LED with 150, 300, and 450 mW incident light power, respectively. The photodegradation of the reactant 4-nitrophenol without Bi NPs was also considered as a background effect in each test. All error bars represent one standard deviation of the mean.

Moving forward to photocatalyzed reactions by Bi nanoparticles, LEDs with different center wavelengths and power intensities were used as the light sources for a wavelength-dependent study. The LEDs are of relatively narrow bandwidths (Figure S5d, e.g., 12.5 nm for 405 nm LED) and are all collimated into a beam size of 0.95 cm, smaller than the 1 cm width of the cuvette used. We are aware that the quantum yield at the same absorbed photon flux is a more rigorous descriptor for comparing the photocatalytic performance across different LEDs.^71,72^ However, due to the light absorption of reactant 4-nitrophenol and the unknown scattering properties of Bi nanoparticles, we choose the incident light power intensities as descriptors when adjusting different wavelengths. Cooling fans and constant stirring were carried out to minimize the photothermal effect as the macroscopic temperature is only expected to rise about 1–2 °C and it should not affect the evaluation of photocatalysis.^45,73^ Under our typical experimental conditions with a 405 nm LED and 150 mW of absorbed power, it is estimated that the temperature rise for each Bi nanoparticle is about 0.00982 °C and the interval time between the two absorbed photons by the same particle is about 5.6 μs (Table S1). As such, the nanoparticle would have already dissipated all the heat long before the time it absorbs another photon. Another background effect in the photocatalyzed reactions is the light absorption of reactant 4-nitrophenol with a peak at around 400 nm in the surveyed region, which might cause the reactants’ photodegradation.^74,75^ Thus, photoreactions without Bi nanoparticles under different LED light irradiations with various power intensities were performed with time-dependent UV–vis profiles in Figure S4 and kinetics analysis in Figure S5. The corresponding initial reaction rate constants are plotted in Figure 3, demonstrating the very limited (about 11% maximum) contributions of photodegradation of the reactants to the respective photocatalyzed reactions. Future ultrafast spectroscopic research needs to be pursued to disentangle the exact mechanism involved in this photodegradation from the overall photocatalysis driven by hot carriers of the nanoparticles.^76,77^ This background effect will be taken into account when comparing the photocatalytic performances across different wavelengths.

As shown in Figure 3, under all different wavelengths of LED irradiations (405, 415, 455, and 530 nm), the apparent rate constants (*k*_app_) increase accordingly with varying increment factors as the incident power increases (150 to 450 mW, see time-dependent profiles in Figure S6). The 405 nm LED light irradiation brings the most significant increase up to more than three times, which aligns with the strong interband absorption at around 400 nm in Figure 1a. The possible underlying pathway is that higher optical power produces more hot electron–hole pairs, and the deep holes from interband transitions are effectively quenched by the excess number of hole scavengers in the reaction solutions. As a result, more electrons are either directly transferred and involved in the reduction process or indirectly accumulated around the Fermi level in the nanoparticles to facilitate the reduction.^14,41^ It is noteworthy that in our reaction conditions, the apparent rate constants at lower incident power (such as 150 mW for 405 and 455 nm LED) are relatively smaller than those under nonirradiation conditions. We speculate that the strong competitive light absorption of reactant 4-nitrophenol with light absorption of Bi nanoparticles might create a different reaction pathway compared to the reaction without light irradiation. This phenomenon of reaction rate suppression under light irradiation was also reported in some other similar hydrogenation reduction reactions by photocatalysis of metallic nanoparticles.^78,79^ When the power intensity increases to a certain extent, the photoexcitation of metallic nanoparticles overwhelms this suppression; thus, a much higher reaction rate occurs. Another note is that the apparent rate constants under 530 nm irradiation are higher than those under 455 nm irradiation, in which we speculate that the cutoff of the 4-nitrophenol absorbance is around 450 nm; thus the 530 nm irradiation will have less competitive light absorption for the nanoparticles and reactants. Hence, when comparing the photocatalytic performance across different wavelengths in the following discussion, we opt to use the rate constants at 150 mW as the threshold baseline to calculate the photocatalysis enhancement factor at higher power intensities.

**Figure 4:**
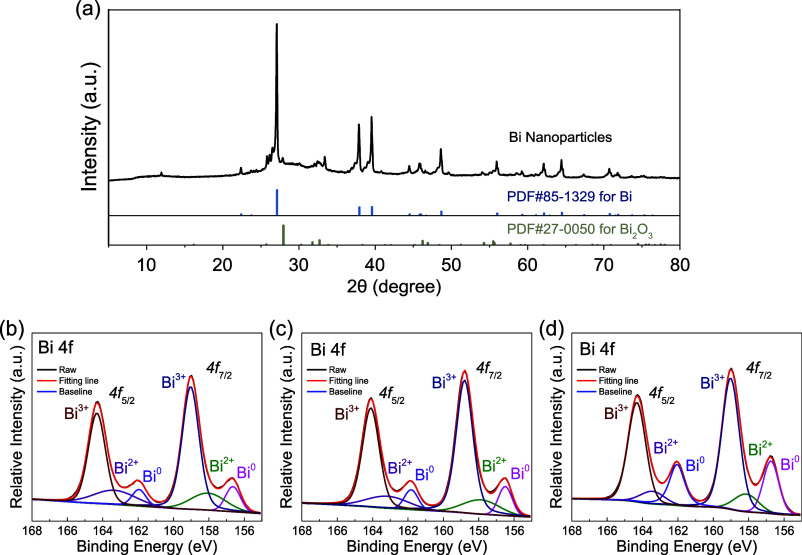
Spectroscopic evidence of the metallic state of Bi nanoparticles. (a) PXRD patterns of as-prepared Bi nanoparticles (in the dried solid form). High-resolution XPS spectra of Bi 4f in (b) as-prepared Bi NPs, (c) Bi NPs after nonirradiation nitrophenol reduction reaction, and (d) Bi NPs after a typical photocatalyzed nitrophenol reduction reaction by 405 nm LED with 450 mW incident power.

Before diving into the mechanistic interpretation, some spectroscopic evidence was collected to confirm the metallic state of Bi nanoparticles for photocatalysis, and some more control experiments were conducted to exclude the contribution of the surface oxide layer to photocatalysis. As shown in Figure 4a, the X-ray diffraction patterns of as-synthesized Bi nanoparticles predominantly match the rhombohedral phase of bismuth metal with partially weak peaks assigned to bismuth oxide. As mentioned in the original synthesis report,^63^ the hydrophobic surface with a layer of sodium oleate capping ligands could be responsible for the stability of metallic Bi nanoparticles. Since the reduction reaction happens on the nanoparticle surface, XPS was also employed to identify the surface status before and after the reactions. In Figure 4b, the high-resolution spectrum of Bi 4f in as-synthesized nanoparticles demonstrates that there is indeed a surface oxide layer containing Bi^3+^ and Bi^2+^, along with about 11% of the metallic Bi^0^ exposed at the surface. After the nonirradiation 4-nitrophenol reduction reaction with NaBH_4_ in Figure 4c, the surface status barely changes with still about 11% metallic Bi^0^. Notably, the amount of metallic Bi^0^ in Bi nanoparticles increases up to 24% after a typical photocatalyzed reaction under 405 nm LED irradiation with 450 mW incident power, as shown in Figure 4d. It is suggested that overwhelmingly produced (or accumulated) electrons from photoexcitation could potentially reconstruct the surface structure to expose more metallic Bi at the surface. A similar phenomenon was also reported in other metallic nanoparticle photocatalysts.^80−82^ Even so, it should not affect our mechanistic interpretation based on the hot carriers, and the surface of Bi nanoparticles should remain in a metallic state during the reaction, considering the strong reducing agent of NaBH_4_ used under all reaction conditions.

To further exclude the possible mechanism of bismuth oxide contribution in photocatalysis, control experiments with comparable sizes of Bi_2_O_3_ nanoparticles were performed under the same nonirradiation and typical photocatalyzed reaction conditions (Figure S7). The Bi_2_O_3_ nanoparticles show almost no activities toward the 4-nitrophenol reduction under nonirradiation conditions and significantly lower activities under 405 nm LED with 450 mW incident power compared to the Bi nanoparticles. No obvious metallic Bi was detected in the high-resolution XPS spectrum of Bi 4f in the Bi_2_O_3_ nanoparticles in Figure S8. Only about 5% of metallic Bi^0^ was found in the Bi_2_O_3_ nanoparticles after nonirradiation and photocatalyzed reactions. All of these findings confirm that the active sites in Bi nanoparticles are in the metallic form, and the hot-carrier-induced mechanism is the only responsible pathway for the enhancement of photocatalyzed reactions.

**Figure 5:**
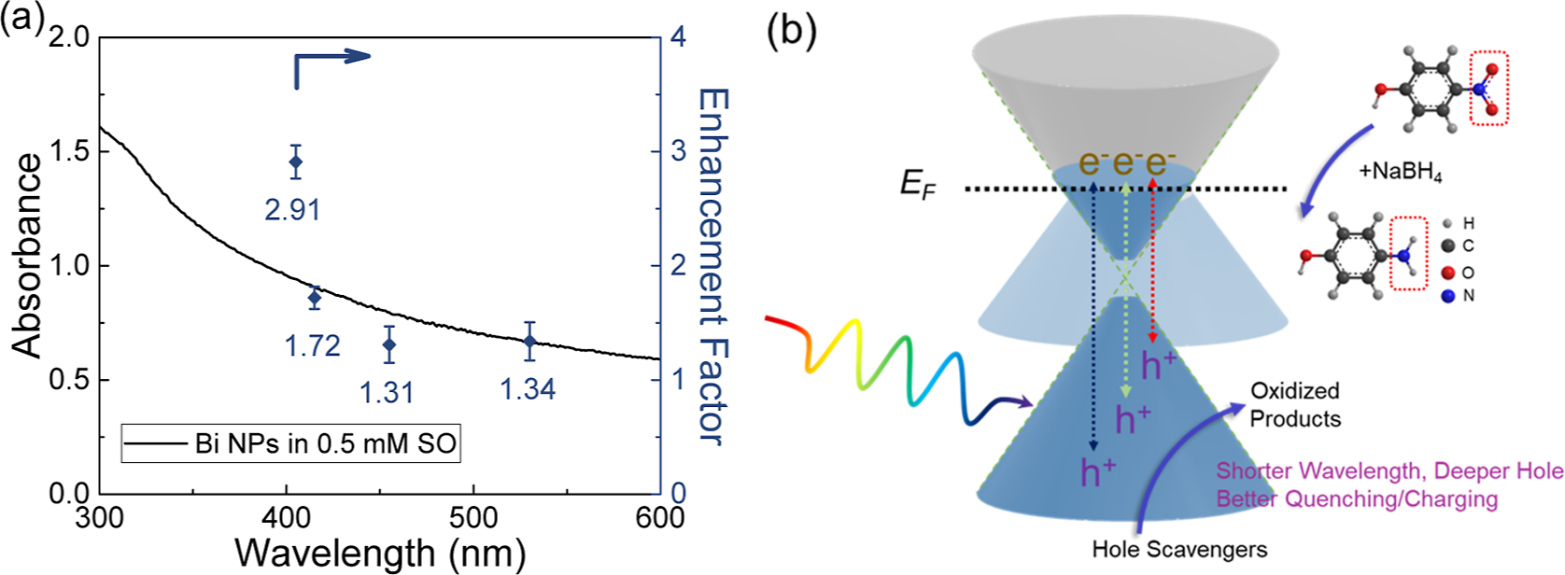
Mechanistic interpretation. (a) Wavelength-dependent photocatalysis enhancement factor of Bi nanoparticles (right axis) compared with their absorbance (left axis). The enhancement factor was calculated by the ratio of reaction rate constants at 450 mW and at 150 mW at each wavelength. (b) Proposed photocatalysis mechanism of Bi nanoparticles, aligned with the interband transitions.

As mentioned earlier, the photocatalysis enhancement factor, defined as the ratio of apparent reaction constants at higher optical power and at 150 mW as the threshold baseline, was selected to compare different wavelengths and avoid any light-induced pathway interference.^83^ The wavelength-dependent enhancement factor of photocatalyzed reactions was plotted with the optical absorption of Bi nanoparticles in Figure 5a for comparison. At shorter wavelengths (405 and 415 nm) with higher photon energy, a significant increase in the reaction rate constants was observed, and the small difference in optical responses at these two wavelengths is insufficient to account for this enhancement. The most probable explanation is that more electrons, either directly transferred from quenching or indirectly accumulated from charging processes, are involved in the reduction reaction at higher optical power levels. Additionally, the enhancement factor remained flattened out at longer wavelengths (455 and 530 nm), probably because the hot holes are not deep enough to be effectively quenched by hole scavengers, thus, limiting the number of electrons involved in the reaction. This wavelength-dependent performance is a signature trend of interband transitions, as observed in other metallic nanoparticle photocatalysts previously, such as Au,^50,84^ Pd,^8,45^ and Co.^25^

At this moment, there might be two different reaction pathways involved in the hot-carrier-induced mechanism, and it is difficult to distinguish between them without any ultrafast spectroscopic insight into the hot carrier’s behavior in Bi nanoparticles, which deserves future studies. The first one is direct electron transfer from the metal to metal–adsorbate unoccupied antibonding states, and the hole scavengers are used to quench the hot holes and increase the electron injection efficiency.^85^ Even the interband transitions only promote the hot electrons around the Fermi level with relatively low potential energy; however, these electrons with high kinetic energy, especially for bismuth with a highly anisotropic Fermi surface, may still have a reasonable chance to travel across the interface of metal–adsorbates states. This pathway may also benefit from the long carrier mean free path and high carrier mobility of bulk bismuth, as mentioned in the Introduction section, which also needs more direct ultrafast evidence in the case of bismuth nanoparticles. The second possible pathway is through an in situ photocharging process during reactions, which have recently been highlighted as an often-overlooked but essential mechanism.^25,41,46,86^ Since interband transitions produce deeper hot holes below Fermi levels, these holes with strong oxidation power can be favorable to be quenched by hole scavengers, thus accumulating ground-state electrons around the Fermi level and raising the Fermi level gradually. These accumulated electrons can either be directly involved in the reduction reaction or potentially weaken the metal–adsorbate interactions. This background effect always occurs during the photocatalysis of metallic nanoparticles, and it is difficult to identify the exact contribution, considering the relatively slow reaction kinetics in this work. Furthermore, more theoretical work is expected to clarify the metal–adsorbate interactions, especially for sp-band-only hybridization in bismuth, to nail down the exact transfer pathway of those electrons from photoexcitation.^14,38^

Lastly, there have been several reports about metallic Bi nanoparticles as the photocatalysts or cocatalysts for photocatalysis,^31−33,87,88^ in which their mechanistic interpretations focus on the LSPR effect and lack insight in interband transitions. We would argue that the observed resonance peaks in the UV–visible region should be coming from the strong interband transitions when multiple electronic transitions simultaneously serve as coupled oscillators and respond to electromagnetic irradiations.^58^ Surface modifications with ligands or a structure change may cause these resonance peaks to shift from UV (observed in our work in Figure 1a) to visible regions, resembling the traditional free-electron-induced LSPR peaks. Essentially, when considering the hot-carrier dynamics and its contribution to the photocatalyzed reactions, the interband transitions should be a more intuitive model to interpret the mechanism, as shown in Figure 5b. The hot electrons in Bi nanoparticles are photoexcited from low-lying electron pockets in the sp band and possibly reside in hole pockets near the Fermi level. At the same time, the direct small band gap around 36 meV could be beneficial for charge separation with a longer lifetime of hot carriers. On the other hand, the residual counterparts, hot holes, remain in the sp band well below the Fermi level and are of high oxidation power to be extracted for photocatalysis. The electronic Fermi energy of bulk Bi was reported to be around 4.22 eV,^89,90^ and the deep holes generated from interband transitions with a 405 nm incident light (photon energy of 3.06 eV) could have the reduction potential as low as 7.28 eV (as compared to vacuum level). Considering the reduction potentials of NaBH_4_ (H_2_BO_3_^–^/BH_4_^–^, −1.24 V)^91^ and of Bi_2_O_3_ (Bi_2_O_3_/Bi, −0.46 V for bulk and −0.40 V for nanoparticles with a diameter of 76 nm)^92^ as compared to the standard hydrogen electrode (SHE), the oxidation layer on the surface of Bi nanoparticles will be removed and reduced to metallic state during the 4-nitrophenol reduction. On the other hand, considering the reduction potential of isopropanol (acetone/isopropanol, 0.76 V vs SHE)^93^ and the lowest reduction of hot hole generated by photoexcitation (2.84 V vs SHE by 405 nm light excitation), the isopropanol as the scavengers could quench the holes efficiently and lower wavelength excitation with higher photon energy should enhance this quenching process. Specifically in Bi nanoparticles, at shorter wavelengths deeper holes are generated by photoexcitation and quenched by hole scavengers. The hot electrons can be either directly transferred to the reactants or indirectly accumulated for further reduction, eventually enhancing the reduction reaction rate and improving the photocatalytic performance.

## Conclusions

In summary, we demonstrated that the photocatalytic performance of colloidal semimetallic Bi nanoparticles was wavelength-dependent under visible light irradiation. The photocatalysis enhancement factor was correlated with the hot carriers’ properties in interband transitions, in which the hot holes played a critical role. At shorter wavelengths, the hot holes can be quenched by hole scavengers more effectively, thus enhancing the quenching or charging effect to facilitate the electron-transfer process, ultimately increasing the reduction reaction rate. The unique band structure of bismuth semimetal and highlighted mechanistic interpretations of interband transitions in this work will potentially expand the field of metallic nanoparticle photocatalysts with more affordable choices.

## Supporting Information Available

Digital images of colloidal Bi nanoparticle solution; TEM images of Bi nanoparticles; UV–vis spectra of control experiments with only parts of reaction reactants and without nanoparticles under photoirradiation; kinetics analysis of 4-nitrophenol photodegradation without Bi nanoparticles; time-dependent UV–vis spectra of photocatalyzed 4-nitrophenol reduction with Bi nanoparticles; control experiments with Bi_2_O_3_ nanoparticles for 4-nitrophenol reduction reaction; surface oxidation analysis of Bi_2_O_3_ nanoparticles before and after catalyzed reactions; and estimation of temperature rise for colloidal Au, Pd, and Bi nanoparticles (PDF)
