# Supplementary material for: Deciphering the Photocatalysis Mechanism of Semimetallic Bismuth Nanoparticles
Source: J Phys Chem C Nanomater Interfaces. 2024 Nov 16;128(47):20118–28. doi: 10.1021/acs.jpcc.4c06136 (PMC11613560; doi:10.1021/acs.jpcc.4c06136)
Supplement: Supplementary file 1 — jp4c06136_si_001.pdf [file jp4c06136_si_001.pdf]

Supporting Information

Deciphering the Photocatalysis Mechanism of Semi-Metallic Bismuth Nanoparticles

Lauren M. Hoffman, Delaney J. Hennes, and Pin Lyu*
Department of Chemistry and Biochemistry, University of North Carolina Asheville, 1 University Heights, Asheville, North Carolina 28804, United States.
*Corresponding Author: plyu@unca.edu

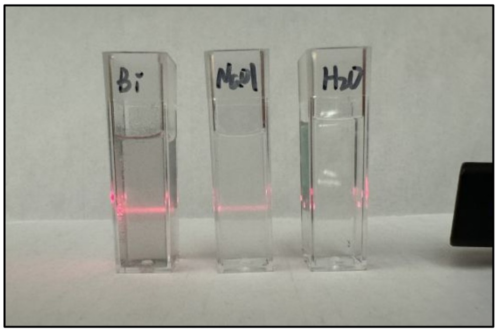

Figure S1. Digital images showing the colloidal form of Bi nanoparticles. From left to right: Bi nanoparticles (stock solution in 0.5 mM NaOl), NaOl solution (0.5 mM) and H_2O as a reference. Since the critical micelle concentration (CMC) of sodium oleate in water is 1 mM,[1] our condition (0.5 mM) should not impact the light absorption of the nanoparticles.[2]

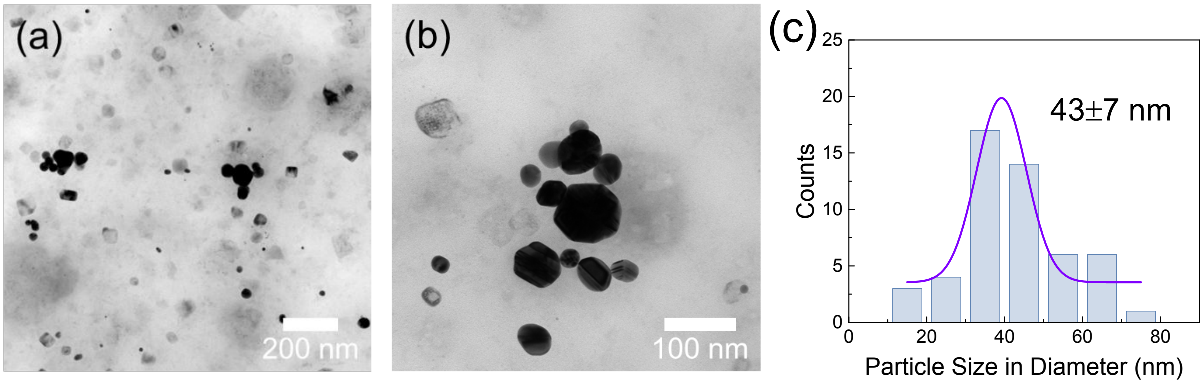

Figure S2. (a, b) TEM images of Bi nanoparticles. The scale bars are 200 nm and 100 nm, respectively. (c) Size distribution analysis of Bi nanoparticles from TEM images with a Gaussian fitting curve. The average diameters of Bi nanoparticles are 43±7 nm (more than 50 particles counted). Note that the aggregations of Bi nanoparticles in panels (a) and (b) were excluded from size determination.

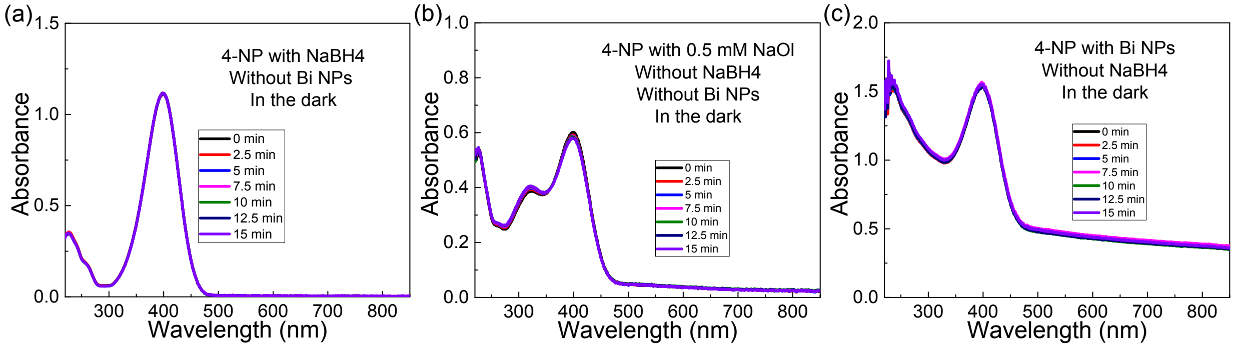

Figure S3. Control experiments to confirm the activity of Bi NPs under non-irradiation conditions. (a) 4-Nitrophenol solution with $NaBH_4$ and without Bi NPs. (b) 4-Nitrophenol solution with 0.5 mM NaOl, without $NaBH_4$ and without Bi NPs. (c) 4-Nitrophenol solution with Bi NPs and without $NaBH_4$. 4-NP represents for 4-Nitrophenol.

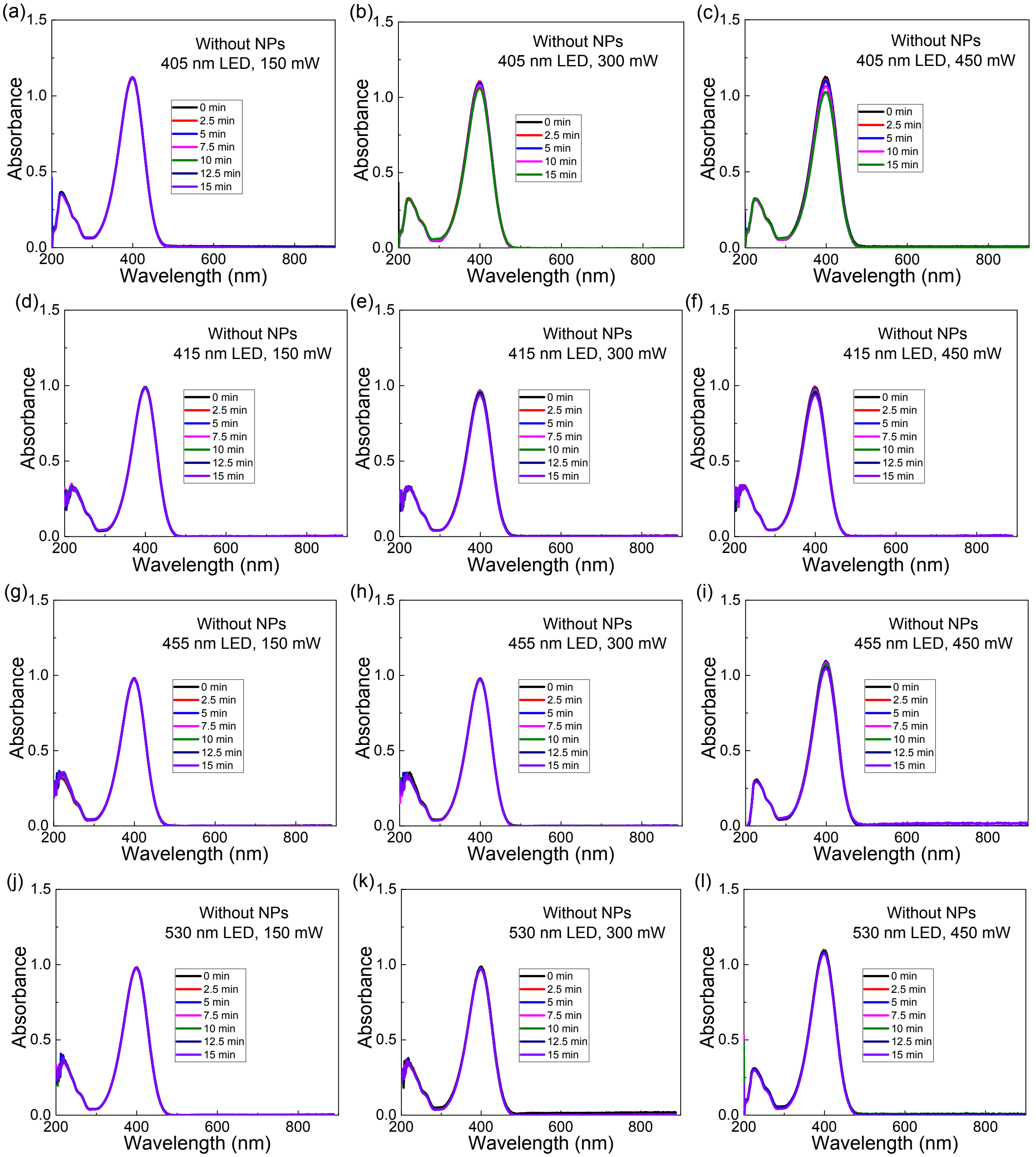

Figure S4. Time-dependent UV-vis profile of 4-nitrophenol photodegradation without Bi NPs under different LED light irradiations. (a, b, c) 405 nm LED, (d, e, f) 415 nm LED, (g, h, i) 455 nm LED, and (j, k, l) 530 nm LED with 150, 300 and 450 mW incident light power.

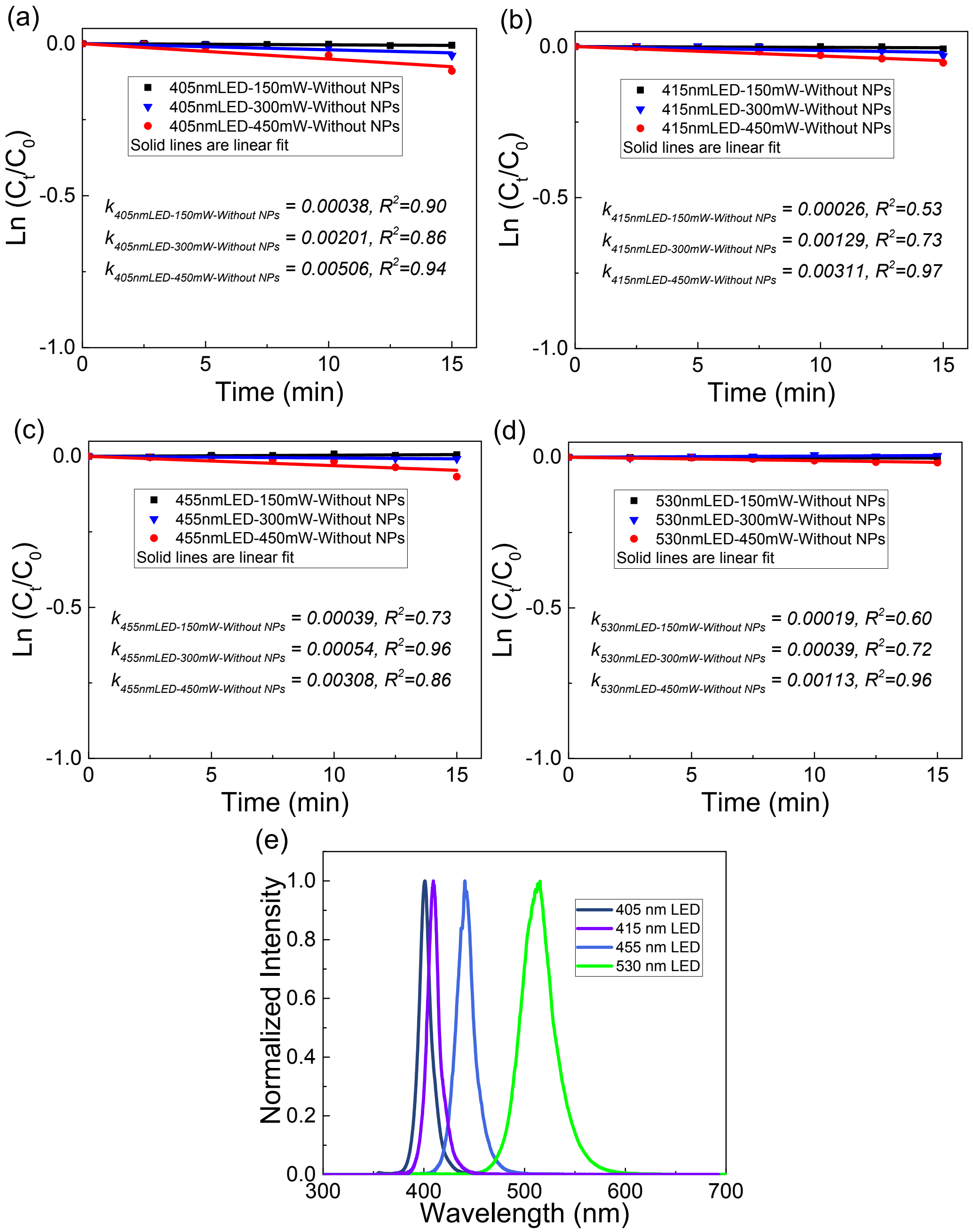

Figure S5. Kinetics analysis of 4-nitrophenol photodegradation without Bi NPs under different LED light irradiations. (a) 405 nm LED, (b) 415 nm LED, (c) 455 nm and (d) 530 nm LED with 150, 300 and 450 mW incident light power. The linear fits follow a pseudo-first-order process for 4-nitrophenol. The apparent rate constants extracted are plotted in Figure 3 in the main text. (e) Normalized intensity of LED light sources used in this work. The bandwidths (full width at half maximum) of 405, 415, 455 and 530 nm LEDs are 12.5, 14, 18, and 35 nm, respectively. The spectra are retrieved from Thorlabs specification sheets.[3]

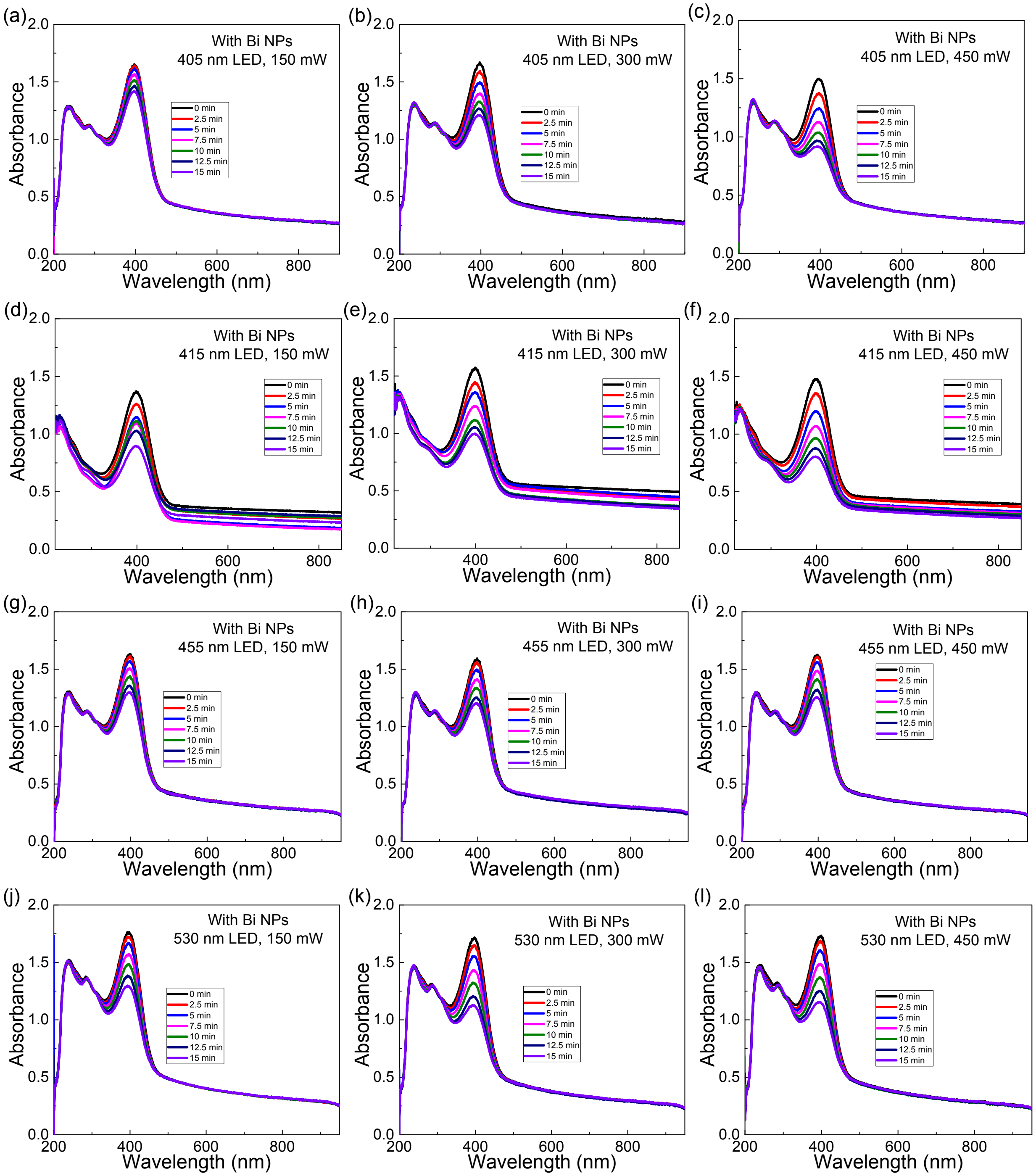

Figure S6. Time-dependent UV-vis profile of 4-nitrophenol reduction with Bi NPs under different LED light irradiations. (a, b, c) 405 nm LED, (d, e, f) 415 nm LED, (g, h, i) 455 nm LED, and (j, k, l) 530 nm LED with 150, 300 and 450 mW incident light power. The kinetics analysis and the linear fits following a pseudo-first-order process for 4-nitrophenol were plotted in Figure 3.

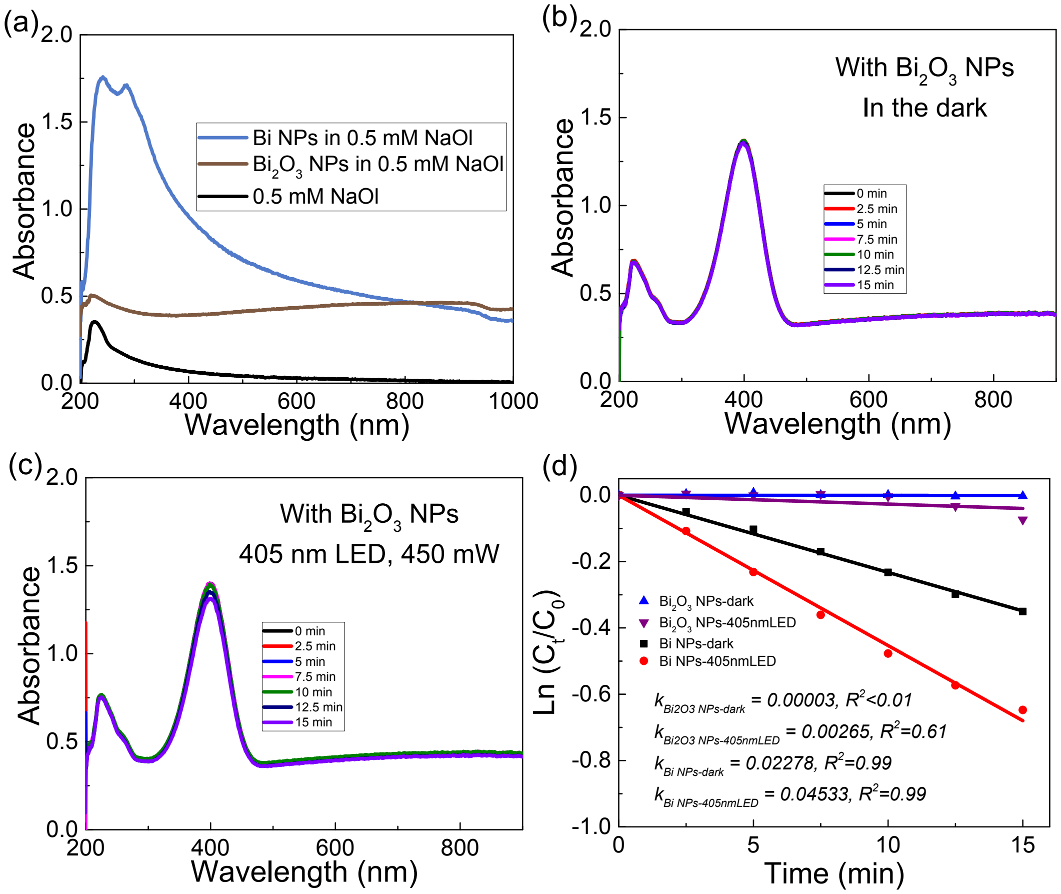

Figure S7. Control experiment with Bi_2O_3 nanoparticles. (a) UV-vis spectra of Bi NPs and Bi_2O_3 NPs in 0.5 mM NaOl (sodium oleate) solution. Time-dependent UV-vis profile of 4-nitrophenol reduction with Bi_2O_3 nanoparticles under (b) non-irradiation and (c) 405 nm LED with 450 mW power light irradiation conditions. (d) Kinetics analysis with Bi_2O_3 nanoparticles for initial apparat rate constant extraction. The linear fits follow a pseudo-first-order process for 4-nitrophenol.

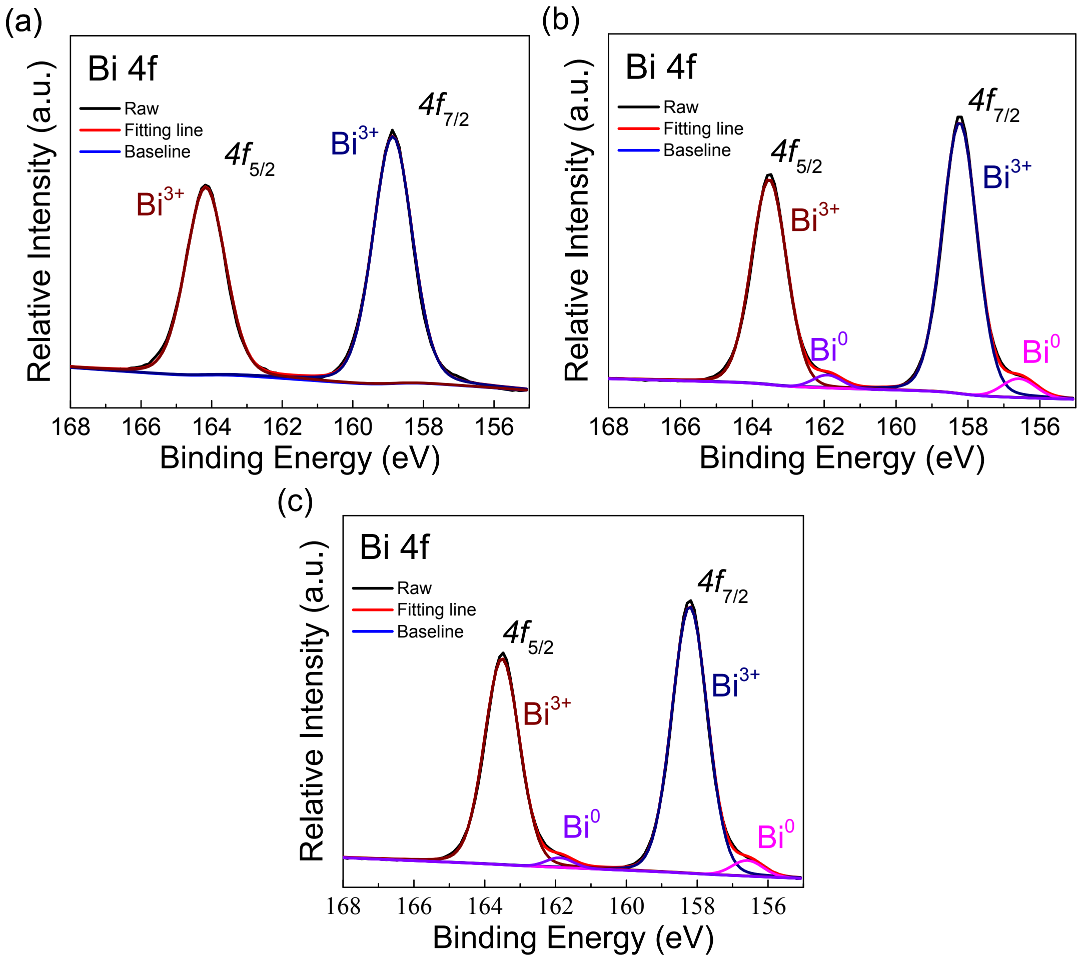

Figure S8. High-resolution spectra of Bi 4f in (a) Bi_2O_3 nanoparticles, (b) Bi_2O_3 nanoparticles after non-irradiation nitrophenol reduction reaction, and (c) Bi_2O_3 nanoparticles after a typical photocatalyzed nitrophenol reduction reaction by 405 nm LED with 450 mW incident power. The metallic Bi were 5 % and 4 % for b and c respectively.

Table S1. Estimation of temperature rise for colloidal Au, Pd and Bi nanoparticles

Type of NPs	Au NPs (spherical)[a]	Pd NPs (mesoporous)[b]	Bi NPs (this work)
Center wavelength of the LED (nm)	405 as an example		
Particle size in diameter (nm)	~41	~63	~43
Concentration of the metal in the reaction solution (mM)	0.3	2	9.38
Amount of light absorbed by the nanoparticle catalysts in the reaction solution (mW)	90	81	150
Number of the photons absorbed by the nanoparticle per second (photon/second)	1.84×10^{17}	1.65×10^{17}	3.06×10^{17}
Volume of the LED beam passed through the reaction solution (mL)[c]	0.708	0.708	0.708
Total number of nanoparticles in the reaction solution (particle)	8.49×10^{10}	1.37×10^{11}	4.83×10^{12}
Total number of nanoparticles in the LED beam path (particle)	3.01×10^{10}	4.84×10^{10}	1.71×10^{12}
Average number of photons absorbed by each nanoparticle per second (photon/(particle·second))	6.11×10^{6}	3.41×10^{6}	1.79×10^{5}
Average time gap between two absorbed photons for each nanoparticle (nanosecond)	164	293	5590
Average temperature rise of each nanoparticle (°C)[d]	5.46×10^{-3}	1.31×10^{-3}	9.82×10^{-3}

(a) Data retried retrieved from reference.[4]
(b) Data and calculation method retrieved from reference.[5]
(c) Volume = $\pi(\frac{\varphi}{2})^2 l$, where: $\varphi =$ 0.95 cm is the LED beam size, and $l =$ 1 cm is the beam path length in the reaction solution.
(d) Average temperature rise = energy of a photon/(specific heat × mass of a single particle), where: the mass of a nanoparticle was calculated based on the particle diameter and density of the metal nanoparticles respectively.

Notes for Table S1: Our experimental condition with low-power continuous-wave LED irradiation and constant stirring differs greatly from other experimental conditions, where high-power pulse lasers were used and heat dissipation was poor. Local heating, the temperature rise in the vicinity of individual nanoparticles in our experiment after a nanoparticle absorbs a photon, should be negligible after considering the three timescales of the three processes. The detailed explanations and calculation methods have been

reported in our previous work,[5] and we only briefly recap some of the critical points here. **The first timescale** is the relaxation time of the hot carriers, which eventually converts the energy of an absorbed photon into heat in the nanoparticle. The nanoparticles should have an even temperature distribution within a very short time (few ps for Au) after the well-thermalization of ultrafast electron-electron and electron-phonon couplings.[6] For bismuth nanoparticles, even though the photophysics is still unknown at this moment, using the same estimated method gives the temperature rise of 0.00982 ^{o}C per nanoparticle. **The second timescale** is the time for the heat transfers from the nanoparticle to the surrounding environment. This value was reported to be around 2-3 ns for gold nanoparticles in water,[7] and it is reasonable to assume that it should be in the same magnitude for Pd and Bi. **The third timescale** is the period between the two photons that can be absorbed by the same nanoparticle. In a typical reaction condition (405 nm LED, 150 mW of absorbed power), this value is estimated to be around 5.6 μs for bismuth nanoparticles in this work, which is much larger than the second timescale above. Thus, the nanoparticle would have already dissipated all the heat long before the time it absorbs another photon.[8] Under our stirring condition, the accumulated heat in the beam path is effectively transferred to the entire cuvette.[9] With a cooling fan, the reaction solution in the cuvette only has a temperature rise within 1-2 ^{o}C.

References:

(1) Merta, J.; Stenius, P. Interactions between cationic starch and anionic surfactants. *Colloid. Polym. Sci.* **1995,** *273,* 974-983.

(2) Mabrouk, M. M.; Hamed, N. A.; Mansour, F. R. Physicochemical and electrochemical methods for determination of critical micelle concentrations of surfactants: a comprehensive review. *Monatshefte für Chemie - Chemical Monthly* **2022,** *153,* 125-138.

(3) Visible LED Spectra Normalized to Max Intensity or Scaled to Min Power. Thorlabs, Inc. https://www.thorlabs.com/newgrouppage9.cfm?objectgroup_id=2692.

(4) Mao, Z.; Vang, H.; Garcia, A.; Tohti, A.; Stokes, B. J.; Nguyen, S. C. Carrier Diffusion—The Main Contribution to Size-Dependent Photocatalytic Activity of Colloidal Gold Nanoparticles. *ACS. Catal.* **2019,** *9,* 4211-4217.

(5) Lyu, P.; Espinoza, R.; Khan, M. I.; Spaller, W. C.; Ghosh, S.; Nguyen, S. C. Mechanistic insight into deep holes from interband transitions in Palladium nanoparticle photocatalysts. *iScience* **2022,** *25.*

(6) Link, S.; El-Sayed, M. A. Shape and size dependence of radiative, non-radiative and photothermal properties of gold nanocrystals. *Int. Rev. Phys. Chem.* **2000,** *19,* 409-453.

(7) Nguyen, S. C.; Zhang, Q.; Manthiram, K.; Ye, X.; Lomont, J. P.; Harris, C. B.; Weller, H.; Alivisatos, A. P. Study of Heat Transfer Dynamics from Gold Nanorods to the Environment via Time-Resolved Infrared Spectroscopy. *ACS Nano* **2016,** *10,* 2144-2151.

(8) Khurgin, J. B. Fundamental limits of hot carrier injection from metal in nanoplasmonics. **2020,** *9,* 453-471.

(9) Un, I.-W.; Sivan, Y. The Role of Heat Generation and Fluid Flow in Plasmon-Enhanced Reduction–Oxidation Reactions. *ACS Photonics* **2021,** *8,* 1183-1190.
